# The benefit of anti-angiogenic therapy in EGFR exon 21 L858R mutant non-small cell lung cancer patients: a retrospective study

**DOI:** 10.1038/s41598-022-18889-z

**Published:** 2022-08-26

**Authors:** Liangkun You, Xinnan Zheng, Danchen Deng, Hongming Pan, Weidong Han

**Affiliations:** 1 Department of Medical Oncology, Sir Run Run Shaw Hospital, Zhejiang University School of Medicine, 3# East Qinchun Road, Hangzhou, 310016 Zhejiang China; 2 Laboratory of Cancer Biology, Key Laboratory of Biotherapy of Zhejiang Province, Sir Run Run Shaw Hospital, Zhejiang University School of Medicine, Hangzhou, Zhejiang China

**Keywords:** Cancer therapy, Lung cancer

## Abstract

Patients with epidermal growth factor receptor (EGFR) exon 21 L858R substitution benefit less from standard EGFR tyrosine kinase inhibitor (TKI) treatment, and whether anti-angiogenic therapy was beneficial to the EGFR L858R subpopulation was inconclusive. A retrospective study was conducted to investigate the survival benefit and the target characteristics of the anti-angiogenic agent in the EGFR L858R patients in our center, comparing those treated with or without anti-angiogenic therapy (cohort A and cohort B). At the median follow-up time of 31.0 months vs 32.7 months (cohort A vs. B) respectively, Cohort A (n = 58) had a significantly prolonged median OS compared to Cohort B (n = 101) (60.0 months vs.37.0 months, HR 0.51, *p* = 0.016). Anti-angiogenic therapy significantly prolonged the OS in patients with liver metastases (NA vs.26.0 months, HR 0.17, *p* = 0.023) comparing to patients without liver metastases (60.0 months vs.37.0 months, HR 0.63, p = 0.129). For brain metastatic patients, anti-angiogenic treatment tended to improve median OS with (65.0 months vs.35.0 months, HR 0.29, *p* = 0.068) or without brain radiotherapy (73.0 months vs.29.0 months, HR 0.24, *p* = 0.171). The grade 3 or more adverse events were manageable and consistent with previous studies. Patients with EGFR L858R mutation treated with anti-angiogenic therapy in their course of treatment had a significantly prolonged OS compared to those who had never received an anti-angiogenic agent. Patients with liver metastases might benefit more from anti-angiogenic therapy than those without.

## Introduction

Despite dashing improvements in countermeasures, lung cancer is still the leading cause of tumor-related death worldwide^1,2^. Epidermal Growth Factor Receptor (EGFR) sensitizing mutation targeted therapy represents one of the breakthroughs in non-small cell lung cancer (NSCLC) treatment in the new century^3^. Most of these mutations appear in adenocarcinoma, while 90% of them are either exon-19 deletions or an exon-21 leucine to arginine substitution (L858R)^4^.

There are three generations of Epidermal Growth Factor Receptor Tyrosine Kinase Inhibitors (EGFR TKIs) designed to target these mutations. Several clinical trials have demonstrated first-generation EGFR-TKIs were associated with an objective response rate (ORR) of approximately 60–80% in patients with EGFR-mutant NSCLCs with a median progression-free survival (PFS) of 8.3–13.1 months^5,6^. While second-generation EGFR-TKI further improved the PFS to 11.0–16.5 months^7,8^. A Phase III clinical trial of the third-generation EGFR-TKI osimertinib had pushed the first line PFS to 18.9 months^9^, with overall survival of 38.6 months^10^. However, in most trials regarding EGFR TKIs, patients with EGFR exon 21 L858R substitution benefit far less from the treatments compared to those with EGFR exon 19 deletion^11,12^. For example, L858R patients treated with the first line osimertinib only had a PFS of 14.4 months, compared to 21.4 months in patients with 19 deletions^9^, with no overall survival benefit over first-generation TKIs^10^. Thus, strategies for overcoming L858R substitution are still calling for attention.

Anti-angiogenic agents had been a competent addition to standard therapy in NSCLC. Starting with the BeTa lung phase 3 study, the combination of bevacizumab and erlotinib was tested in patients of NSCLC without EGFR mutation stratification, and subgroups analyses showed patients with EGFR-positive NSCLC responded better to the combination^13^. The JO25567 phase 2 trial was then initiated and saw a significant improvement of median PFS in the erlotinib plus bevacizumab group compared with the erlotinib alone (16.0 months [95% CI 13.9–18.1] vs 9.7 months [5.7–11.1], hazard ratio [HR] 0.54 [95% CI 0.36–0.79]) with an acceptable toxicity profile^14^. NEJ026 was a phase 3 clinical trial aiming to validate the result of the JO205567, a median PFS of 16.9 months (95% CI 14.2–21.0) was seen in patients of the erlotinib plus bevacizumab group compared with 13.3 months (95% CI 11.1–15.3) in patients of the erlotinib monotherapy group (HR 0.605, 95% CI 0.417–0.877). Interestingly, subgroup PFS analysis of NEJ026 showed only those with exon 21 L858R mutation favored the combination, but not the exon 19 deletion patients^15^. However, the recent survival updates of both studies failed to reveal any significant impact on overall survival (OS), despite numerical advantages^16^. The RELAY study and the most recent ARTEMIS-CTONG1509 study had a similar result with PFS in the L858R subgroup, but the OS data of both trials remain immature to date^17,18^. Notably, bevacizumab treatment beyond progression with chemotherapy has been investigated in a randomized phase 3 Avastin in All Lines Lung (AvaALL) trial in NSCLC^19^. Data from the AvaALL study indicated that there might be some advantages from treatment with bevacizumab across multiple lines. The REVEL study tested the addition of ramucirumab to second-line docetaxel treatment. Patients with previous bevacizumab treatment were enrolled in the study, significant PFS and OS were both reached^20^, indicating anti-angiogenic therapy may have a benefit after progression. However, the benefit of anti-angiogenic therapy beyond anti-angiogenesis plus EGFR-TKI (A + T) resistance had not been shown in a clinical study, though frequently seen in real-world practice.

Based on previous evidence, and a lack of data for the benefit of anti-angiogenic agents used across multiple lines of treatments in the EGFR L858R mutant patients, we conducted a retrospective study that sought to investigate the survival benefit and the target characteristics of the anti-angiogenic agent in the mentioned population.

## Results

### Clinical characteristics

A total of 159 patients with baseline EGFR exon 21 L858R mutated NSCLC met the study’s inclusion criteria were enrolled in the study at our center (Cancer center, Sir Run Run Shaw Hospital, Zhejiang University School of Medicine, Hangzhou, China) between Jan 1, 2015, to Jan 1, 2021. 58 patients received anti-angiogenic therapy during their course of treatment. 101 patients never received any systemic anti-angiogenic agent. Baseline patient clinical characteristics are listed in Table 1, including age, gender, smoking history, ECOG score, Stage (III/IV), histological type, concurrent mutations, brain metastases, liver metastases, and TKI received. There were no differences in clinical characteristics between patients treated with or without anti-angiogenic therapy (cohort A and cohort B). For patients in cohort A who had received anti-angiogenic therapy, 16 patients received A + T in the first line, 12 in the second line, 5 in the third line, and 1 in the fourth line, respectively. The others received anti-angiogenic agents after standard EGFR TKI treatment. Seven patients received crossline anti-angiogenic therapy after progression. For the variety of anti-angiogenic agents, 42 patients received bevacizumab, 14 received apatinib, and 11 received anlotinib. Patients may receive more than one anti-angiogenic agent during their treatment course. Of the 23 patients who had concurrent mutations in cohort A and the other 23 in cohort B. One had 1st line T790M, 20 had 2nd line T790M, 1 had 1st line TP53, and 1 had 1st line BRAF mutation in cohort A; Four had 1st line T790M, 14 had 2nd line T790M, 1 had 1st line S768I, 1 had 1st line L861Q, 1 had 1st line PI3K, 1 had 2nd line MET, and 1 had 1st line HER2 in cohort B.

**Table 1:** Patient characteristics at baseline.

Characteristic	Patients, no. (%)	
	Cohort A	Cohort B
**Clinical characteristics**		
No. of patients	58	101
Median age, years (range)	66 (47–84)	64 (43–94)
Gender		
Male	33 (57%)	41 (41%)
Female	25 (43%)	60 (59%)
Histology		
Adenocarcinoma	55 (95%)	96 (95%)
Adenosquamous carcinoma	1 (2%)	3 (3%)
Not otherwise specified	2 (3%)	2 (2%)
Smoking history		
Never	44 (76%)	77 (76%)
Former	14 (24%)	24 (24%)
ECOG		
0	32 (55%)	68 (67%)
1	26 (45%)	33 (33%)
Stage		
III	4 (7%)	6 (7%)
IV	54 (93%)	95 (94%)
Concurrent mutations		
Yes	23 (40%)	23 (23%)
No	35 (60%)	78 (77%)
T790M mutation		
Yes	21 (36%)	18 (18%)
No	37 (64%)	83 (82%)
Brain metastases		
Yes	18 (31%)	31 (31%)
No	40 (69%)	70 (69%)
Liver metastases		
Yes	9 (15%)	16 (16%)
No	49 (85%)	85 (84%)
EGFR TKI received		
Afatinib	2 (3%)	1 (1%)
Erlotinib	3 (5%)	5 (5%)
Gefitinib	23 (40%)	27 (27%)
Icotinib	30 (52%)	65 (64%)
Osimertinib	0 (0%)	3 (3%)

### Cox regression analyses of overall survival in all eligible

Cox regression was performed in all eligible patients to discriminate factors associated with OS. In univariate analyses, anti-angiogenic treatment (HR 0.51, 95% CI 0.29–0.88, *p* = 0.016) was associated with a reduced risk of death, and the female gender (HR 1.81, 95% CI 1.10–2.98, *p* = 0.019) was associated with an increased risk of death in L858R mutated patients. In multivariate analyses, only anti-angiogenic treatment (HR 0.52, 95% CI 0.29–0.94, *p* = 0.030) remained to be a positive predictor of OS (Table 2).

**Table 2:** Cox regression overall survival analysis.

	Univariate analysis			Multivariate analysis		
	HR	95% CI	*p* value	HR	95% CI	*p* value
ECOG	0.96	0.59–1.56	0.869	1.16	0.67–2.00	0.548
Age > 65	0.81	0.50–1.31	0.394	0.79	0.47–1.33	0.376
Smoking history	0.72	0.42–1.48	0.463	0.80	0.35–1.79	0.580
Gender	1.81	1.10–2.98	0.019	1.76	0.91–3.40	0.090
Brain metastasis	1.14	0.69–1.87	0.620	1.22	0.72–2.06	0.463
Liver metastasis	0.96	0.52–1.75	0.882	1.08	0.58–2.03	0.805
Anti-angiogenesis	0.51	0.29–0.88	0.016	0.50	0.27–0.91	0.025
T790M	0.98	0.58–1.64	0.932	1.19	0.68–2.08	0.548

### Overall survival analyses in all eligible patients

**Figure 1:**
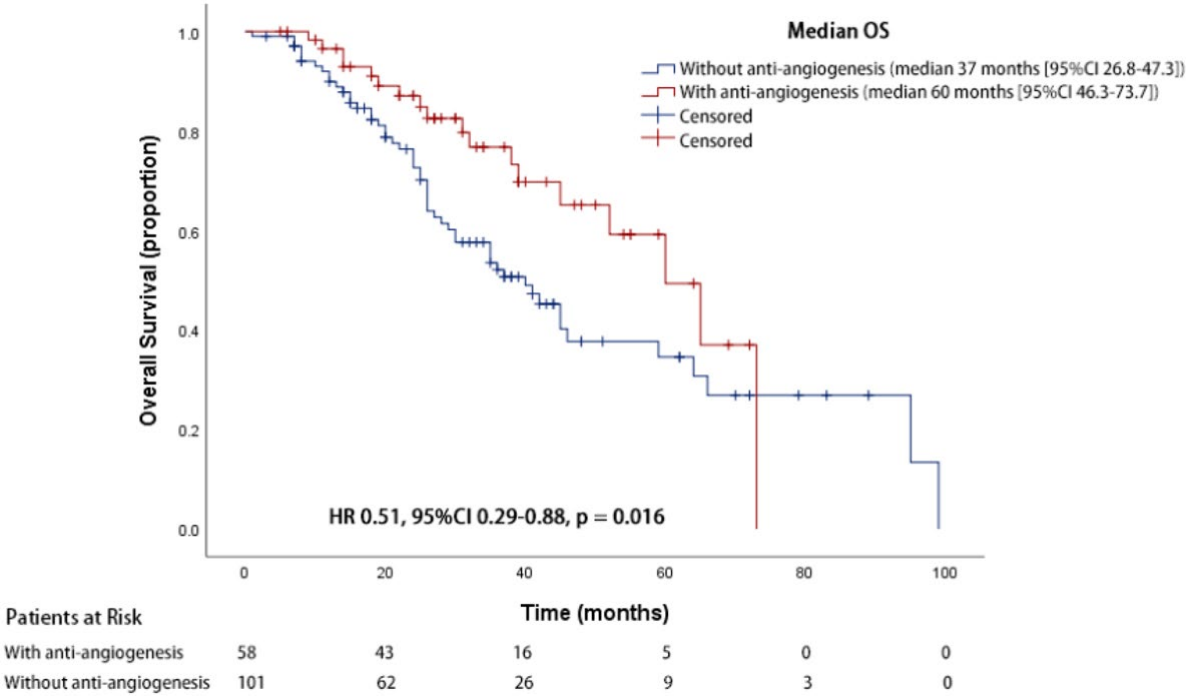
Kaplan–Meier survival curve of overall survival for patients treated with or without anti-angiogenesis therapy.

**Figure 2:**
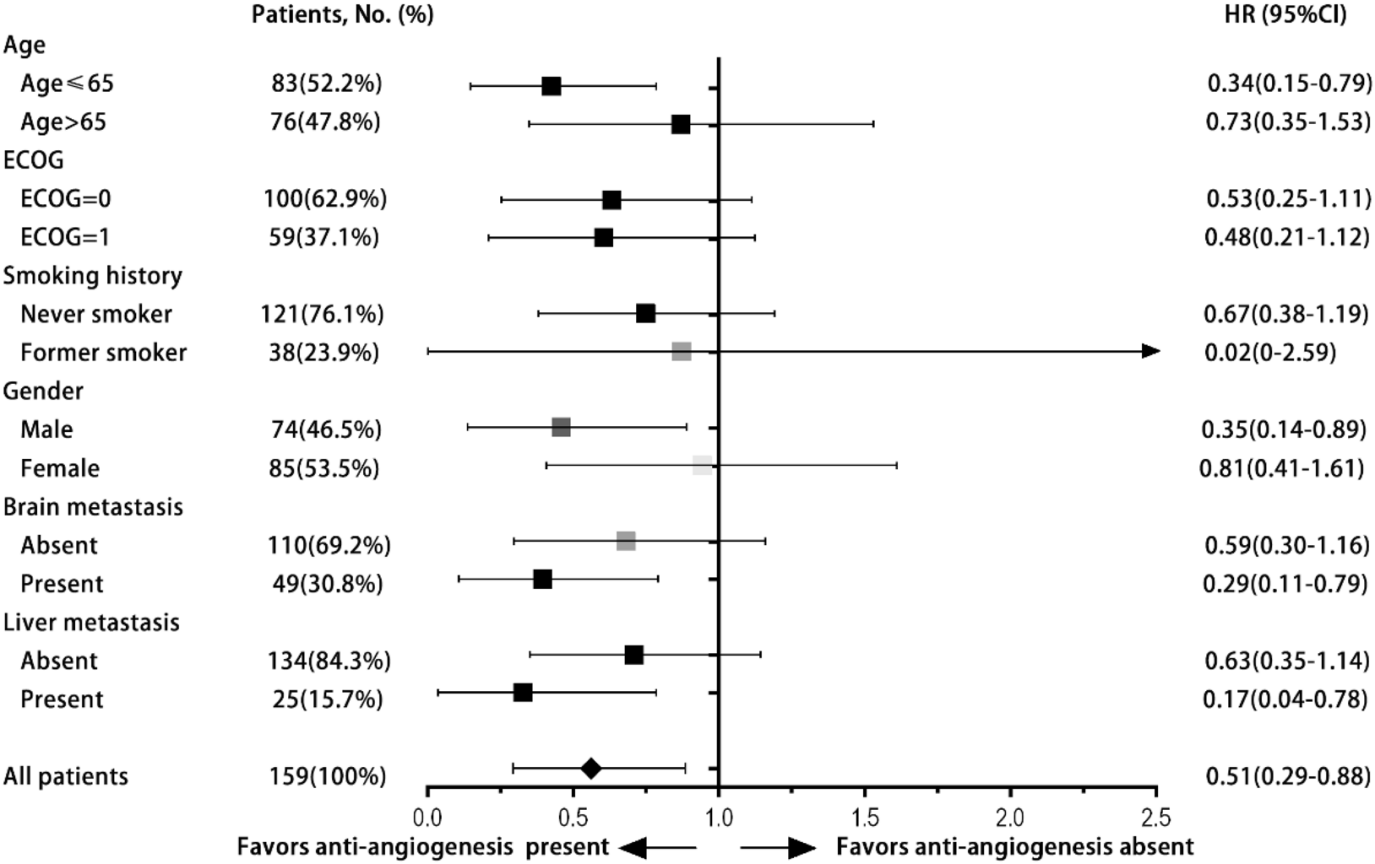
Forest plot of subgroup analysis of OS between patients treated with or without anti-angiogenic therapy.

At the median follow-up time of 31.0 months vs 32.7 months (cohort A vs. B) respectively, Cohort A had a significantly prolonged OS compared to Cohort B (60.0 months vs.37.0 months, 95% CI 46.3–73.7 months for cohort A, 95% CI 26.8–47.3 months for cohort B, HR 0.51, 95% CI 0.29–0.88, *p* = 0.016) (Fig. 1). Subgroup OS analysis suggested survival benefits of anti-angiogenic treatment in patients of 65 and younger (HR 0.34, 95% CI 0.15–0.79, *p* = 0.012), male patients (HR 0.35, 95% CI 0.14–0.89, *p* = 0.027), patients with brain metastasis (HR 0.29, 95% CI 0.11–0.79, *p* = 0.016), and patients with liver metastasis (HR 0.17, 95% CI 0.04–0.78, *p* = 0.023) (Fig. 2).

### Overall survival analyses in patients with liver metastasis

**Figure 3:**
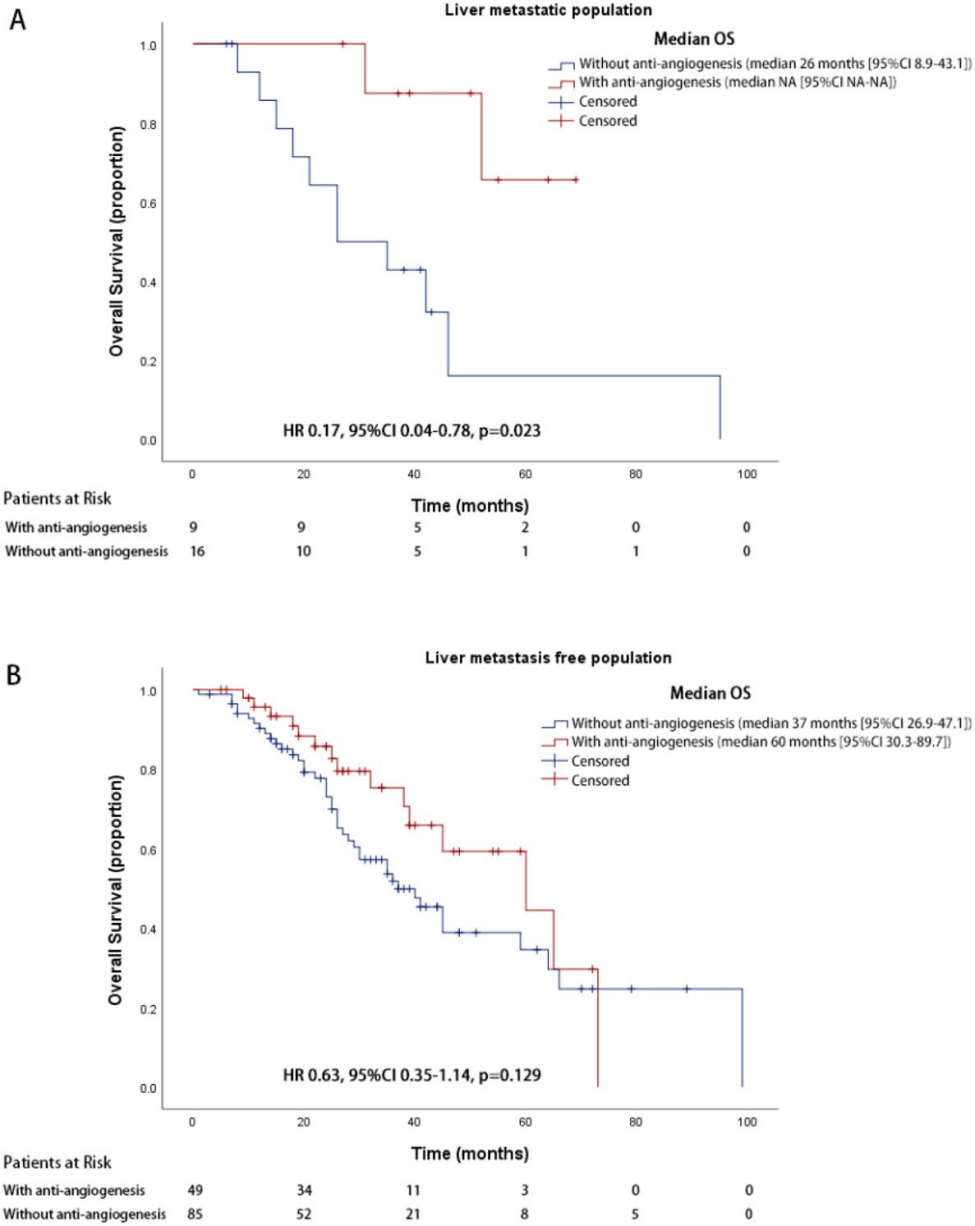
Kaplan–Meier survival curve of overall survival for liver metastatic patients (**A**) or liver metastasis-free patients (**B**) treated with or without anti-angiogenesis therapy.

The OS of patients treated with or without anti-angiogenic therapy by the status of baseline liver metastases was analyzed. OS was significantly improved in Cohort A compared to Cohort B (NA vs.26.0 months, 95% CI NA–NA for cohort A, 95% CI 8.9–43.1 months for cohort B, HR 0.17, 95% CI 0.04–0.78, *p* = 0.023), while the comparison without liver metastasis was not significant (60.0 months vs.37.0 months, 95% CI 30.3–89.7 months for cohort A, 95% CI 26.9–47.1 months for cohort B, HR 0.63, 95% CI 0.35–1.14, *p* = 0.129) (Fig. 3). Patient characteristics are listed in Supplemental Table 1.

### Overall survival analyses in patients with brain metastasis

**Figure 4:**
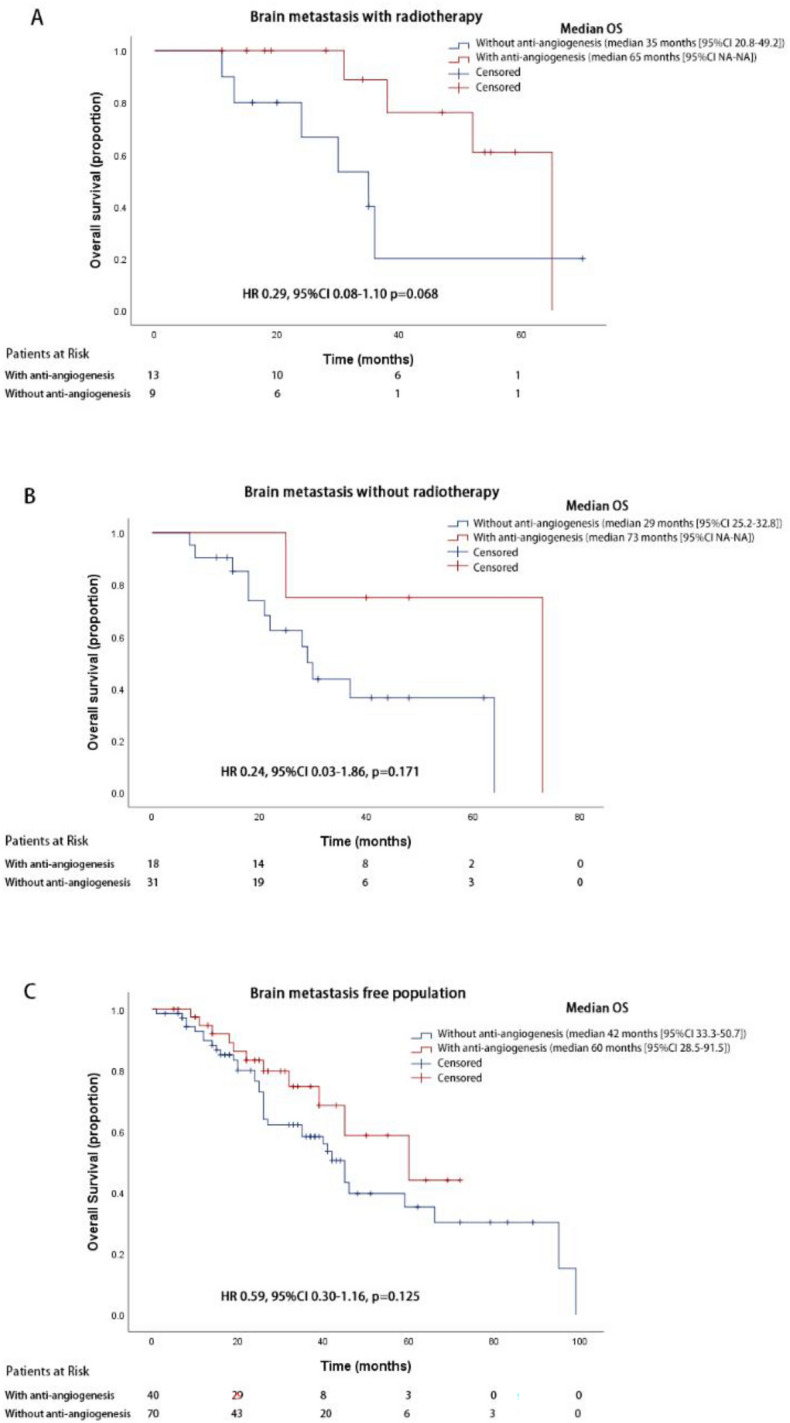
Kaplan–Meier survival curve of overall survival for brain metastatic patients with brain radiotherapy (**A**), brain metastatic patients without brain radiotherapy (**B**), brain metastasis-free patients (**C**) treated with or without anti-angiogenesis therapy.

For OS analyses of patients with baseline brain metastases, brain radiotherapy was used as a stratification factor, and anti-angiogenic therapy tended to benefit all patients subgroups although no statistical significance has been reached. Anti-angiogenic therapy tended to prolong median OS in patients with brain metastases who have undergone brain radiotherapy (65.0 months vs.35.0 months, 95% CI NA–NA for cohort A, 95% CI 20.8–49.2 months for cohort B, HR 0.29, 95% CI 0.08–1.10, *p* = 0.068), and those who have not received brain radiotherapy (73.0 months vs.29.0 months, 95% CI NA–NA for cohort A, 95% CI 25.2–32.8 months for cohort B, HR 0.24, 95% CI 0.03–1.86, *p* = 0.171). Brain metastasis-free patients also tended to survive longer with anti-angiogenic therapy (60.0 months vs.42.0 months, 95% CI 28.5–91.5 months for cohort A, 95% CI 33.3–50.7 months for cohort B, HR 0.59, 95% CI 0.30–1.16, *p* = 0.125) (Fig. 4). Patient characteristics are listed in Supplemental Table 2.

### Overall survival analyses in patients with early-line anti-angiogenesis

**Figure 5:**
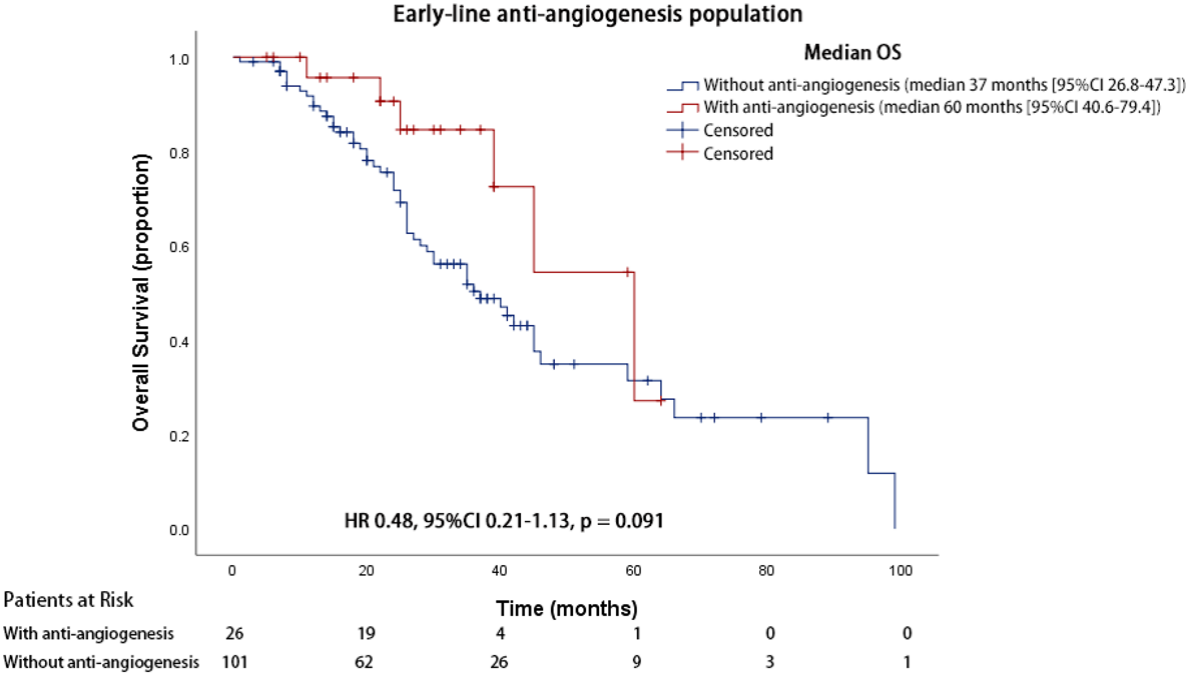
Kaplan–Meier survival curve of overall survival for patients treated with 1st and 2nd line anti-angiogenesis or without anti-angiogenesis therapy.

To avoid selection bias from the inclusion of later-line anti-angiogenic therapy in prolonged survival patients, the OS of patients treated with only the 1st and the 2nd line A + T (cohort C) was compared with patients treated without anti-angiogenic therapy (cohort B). In accordance with the results of cohort A vs cohort B, patients in cohort C also had a longer OS than those in cohort B (60.0 months vs.37.0 months, 95% CI 40.6–79.4 months for cohort C, 95% CI 26.8–47.3 months for cohort B, HR 0.48, 95% CI 0.21–1.13, *p* = 0.091) (Fig. 5). Patient characteristics are listed in Supplemental Table 3.

### Adverse events

45 (78%) of 58 patients in cohort A and 65 (64%) of 101 patients in cohort B had grade 3 or worse adverse events (Table 3). The safety profile is consistent with previous reports. The adverse effects of the combination appeared to be more toxic than monotherapy, especially for hypertension and increased aminotransferase, but were mostly tolerable. Grade 3–4 hypertension was seen in 10 (17.2%) of 58 patients in cohort A and 3 (3.0%) of 101 patients in cohort B. 5 (8.6%) of 58 patients in cohort A and 3 (3.0%) of 101 patients in cohort B had increased aminotransferase. Grade 3–4 rash was common in both groups (6 [10.3%)] of 58 patients in cohort A and 11 [10.9%] of 101 patients in cohort B). There were 8 (13.8%) of 58 patients in cohort A and 16 of 101 (15.8%) patients in cohort B who had Grade 3–4 emesis resulting from combining chemotherapy. No grade 5 adverse events or treatment-related deaths occurred in either group.

**Table 3:** Grade 3–4 adverse effect.

	Cohort A (n = 58)	Cohort B (n = 101)
Acute renal failure	1 (1.7%)	2 (2.0%)
Anemia	1 (1.7%)	4 (4.0%)
Decreased appetite	0	8 (7.9%)
Diarrhea	1 (1.7%)	5 (5.0%)
Emesis	8 (13.8%)	16 (15.8%)
Headache	2 (3.4%)	1 (1.0%)
Hypertension	10 (17.2%)	3 (3.0%)
Increased aminotransferase	5 (8.6%)	3 (3.0%)
Interstitial lung disease	0	1 (1.0%)
Leukocytopenia	2 (3.4%)	3 (3.0%)
Pericardial effusion	0	5 (5.0%)
Pleural effusion	1 (1.7%)	0
Sepsis	1 (1.7%)	0
Rash	6 (10.3%)	11 (10.9%)
Stomatitis	5 (8.6%)	3 (3.0%)
Thrombopenia	2 (3.4%)	2 (2.0%)

## Discussion

The EGFR exon 21 L858R population constitutes almost half of the sensitive mutation patients, but multiple clinical trials and meta-analyses had revealed that EGFR exon 21 L858R benefited less from EGFR-TKIs compared to exon 19 deletion regarding PFS and OS^11,21^. The phase 3 trial NEJ026 comparing the first-line bevacizumab + erlotinib vs erlotinib revealed that median PFS was significantly superior in patients with L858R in the combination group than in the erlotinib group (17·4 months vs 13·7 months, HR 0.57 95% CI 0.33–0.97), a significance that was not reached in those with 19 deletions (16.6 months vs 12.4 months, HR 0.69, 95% CI 0.41–1.16)^15^ but this PFS benefit did not translate into an OS improvement with the 2020 ASCO meeting updates. Subsequent analysis by the authors suggested the additional effect of bevacizumab on erlotinib monotherapy for NSCLC with EGFR mutations gradually decreased in the order of PFS2 (28.6 months vs 24.3 months, HR 0.80; 95% CI 0.59–1.10) and overall survival (50.7 months vs 46.2 months, HR 1.00 95% CI 0.68–1.48), with no significant differences. No survival data by mutation subtype was revealed in the follow-up of the trial. Several meta-analyses had been done to confirm the rationale of this A + T combination^22–24^, however, there has not been a study that succeeded in showing an OS benefit for the A + T combo. The lack of an OS benefit might be explained by the prolonged survival of EGFR mutant patients. Secondly, the crossover of anti-angiogenic therapy after progression may render the first line PFS benefit effectless after several lines of combined therapy.

In our study, we first tried to see if the A + T combo would prolong first-line PFS and translate into an OS benefit. Due to the limited number of cases receiving A + T (n = 16) in the first line, we did not see any statistical significance in either PFS (HR 0.90; 95% CI 0.53–1.54) or OS (HR 0.53; 95% CI 0.17–1.70) for first-line A + T. Seeing anti-angiogenic agents are used in many cases across several lines of treatments in clinical practice in our center and others without enough evidence to support this application, we moved forward to compare the survival data of L858R mutant patients who had received anti-angiogenic therapy in their course of treatment to those who had not and found a significant improvement (60.0 months vs.40.0 months, HR 0.56, p = 0.033). In the NEJ026 trial, the median OS was 50.7 months (95% CI 37.3 months-not reached) with the combination and 46.2 months (95% CI 38.2 months-not reached) with erlotinib alone (hazard ratio, 1.00; 95% CI 0.68 to 1.48).

Historical data suggested patients with liver or brain metastases were especially resistant to standard EGFR TKI treatment^21,25,26^. Liver metastases represent a poor prognosis feature in NSCLC patients, and data indicating optimal systemic therapy for liver metastatic tumors has been scarce^4,26,27^. In the RELAY study, subgroup analysis suggested the addition of ramucirumab may improve PFS in patients with or without liver metastases at baseline (HR 0.48 vs 0.65)^17^. Our result reiterated the benefit of anti-angiogenic therapy in L858R patients with liver metastases. (NA vs.26.0 months, HR 0.17, 95% CI 0.04–0.78, p = 0.023), comparing to patients without liver metastases (60.0 months vs.41.0 months, HR 0.70, 95% CI 0.39–1.24, p = 0.218). This difference in improvements calls for more investigations with larger sample size and prospective design to confirm. Osimertinib was the EGFR-TKI with superior efficacy in brain metastatic patients, the subgroup analysis of the FLAURA trial showed that median CNS PFS was not reached with osimertinib and 13.9 months with first-line EGFR-TKI (HR 0.48; 95% CI 0.26–0.86; p = 0.014)^28^. However, whether brain metastatic patients with L858R mutation were as susceptible as the others is still undetermined. Dacomitinib had the best PFS and OS data in L858R patients^8^, but patients with brain metastases were excluded from the ARCHER 1050 study, thus evidence-based data is still missing for dacomitinib in this subgroup of patients. Several meta-analyses had polled the data from the existing studies comparing A + T vs standard EGFR-TKI, benefit was seen in the A + T group despite brain metastasis^22^. Especially, in the ARTEMIS-CTONG 1509 study, bevacizumab in combination with erlotinib significantly prolonged the PFS of patients with brain metastases, from 11.1 months (95% CI 9.7–12.5) to 17.9 months (95% CI 15.2–20.7) (HR 0.48; 95% CI 0.27–0.84; p = 0.008), as well as OS, from 26.8 months (95% CI 19.5–32.6) to 31.6 months (95% CI 23.0–44.3) (HR 0.62; 95% CI, 0.38–1.01; p = 0.052)^18^. However, there has not been any conclusion drawn regarding L858R mutant patients with brain metastases. In our study, we demonstrated that all patient subgroups, with or without brain metastases, and no matter the case of brain radiotherapy, all tended to fare better with anti-angiogenic therapy in their course of treatment. Generally, brain metastatic patients had a larger difference in survival, with brain radiotherapy (65.0 months vs.35.0 months; HR 0.29, 95% CI 0.08–1.10) or not (73.0 months vs.29.0 months; HR 0.24, 95% CI 0.03–1.86), comparing to brain metastases free patients (60.0 months vs.42.0 months, HR 0.59, 95% CI 0.30–1.16). Coinciding with the findings in the CTONG 1509 study, although OS for patients with brain metastases was numerical longer, we did not see a statistical significance.

Furthermore, we considered the possibility of prolonged survival patients might have higher chances of receiving anti-angiogenic agents, leading to a selection bias resulting in longer survival in cohort A. We first did a comparison between the lines of therapy received in cohort A and cohort B. Cohort A had an average of 3.8 lines of therapy while cohort B had an average of 2.2 lines of therapy, which was significantly fewer. Thus we excluded the later-line patients treated with anti-angiogenic therapy and only compared patients treated with 1st and 2nd line A + T (cohort C) with those who were treated without anti-angiogenic therapy (cohort B) and the result showed patients in cohort C survived 23 months longer than cohort B (60.0 months vs.37.0 months, HR 0.48, *p* = 0.091), which was numerically the same as cohort A comparing to cohort B (60.0 months vs.37.0 months, HR 0.51, *p* = 0.016). However, since the number of patients in cohort C was only 26, distinctly smaller than cohort A which was 58, thus the difference was statistically insignificant (*p* = 0.091 vs *p* = 0.016). We also compared the lines of treatment in cohort C and cohort B, which was 3.0 vs 2.2. In our opinion, this discrepancy may be explained in both ways: On one hand, patients who received 1st and 2nd line anti-angiogenic therapy may genuinely have better survival and was able to receive more lines of therapy; On the other hand, lines of therapy may, in fact, be a confounding factor in this study which calls for a prospective design to overcome.

There is a multitude of reasons why tumors with L858R substitution fare worse than those with exon 19 deletion. The L858R mutation locates at the A-loop of EGFR’s C-lobe, closer to the activation loop of the kinase domain. The substitution destabilizes inactive EGFR tyrosine kinase conformation and stabilizes the active conformation. TKIs generally had a lower affinity with L858R than 19 deletions, hence lower sensitivity^29^. Differences in the phosphorylation of downstream ERK and AKT pathways were also observed^30^. There were reports showing tyrosine 845 is highly phosphorylated in the L858R mutant, leading to an upregulation of the STAT3 cell survival pathway^31^. Interestingly, the L858R mutant also tends to have a poorer immune profile, represented by a higher tumor mutation burden and upregulation of the CXCL12-CXCR4 chemokine receptor pathway^32,33^. However, whether an interaction between L858R tyrosine kinase activation and the VEGFR pathway exists or not remains to be elucidated.

Based on previous trial data, there are a few drawbacks to the A + T combination. One of them is the T790M positive rate. In the ARTEMIS trial, patients from the combination-arm developed fewer acquired T790M resistance when progressed (41% in combination vs 61% in monotherapy)^18^. The same trend was observed in the RELAY trial, with respectively 25% vs 30% T790M mutation for the combination and monotherapy groups^17^. This loss of second-line breaches may be countered by saving anti-angiogenic agent for later lines or combining it with 3rd generation TKI, but trials to answer this specific question are still ongoing.

The present study has a few limitations. First, this was a retrospective design study from a single center. Ideally, it would be better to control the anti-angiogenic treatment in just the first and second line, and evaluate PFS1, PFS2, and OS, respectively, but limited by the numbered of cases receiving A + T in each line, we were unable to independently evaluate the addition of anti-angiogenic treatment in each line of therapy. Second, since we aim to investigate anti-angiogenic in not only L858R patients receiving first-line treatment but also at later lines, thus patients who survived longer may have a better chance to receive therapy, However, we tried to limit this shortcoming by doing an analysis excluding patients receiving later-line anti-angiogenic agent. The result echoed our initial finding that L858R mutant patients might benefit from anti-angiogenic therapy. Even though this was a real-world study with limitations we have uncovered clues for the benefit of anti-angiogenic therapy across all lines of treatment and underlined its possible target patients. The questions of when and how and to whom with EGFR L858R substitution should we add the anti-angiogenic agent are still inconclusive, thus warranting more efforts for the benefit of this specific mutation population.

In conclusion, this study demonstrated that NSCLC patients who harbored L858R substitution treated with anti-angiogenic therapy have better overall survival, especially for those with liver or brain metastasis.

## Patients and methods

### Patients

All NSCLC patients with EGFR exon 21 L858R substitution who received first-line EGFR-TKI were enrolled in this study for survival analysis. The main inclusion criteria were as follows: unresectable stage III/IV (according to the 8th American Joint Committee on Cancer Staging System), histologically confirmed non-squamous NSCLC with EGFR exon 21 L858R mutation, at least one radiological response evaluation according to Response Evaluation Criteria in Solid Tumors (RECIST) V1.1, treated with first-line EGFR-TKI, aged over 18 years old. The main exclusion criteria included previous EGFR-TKI treatment, adjuvant use of EGFR-TKI, and diagnosed concomitantly with other cancers besides NSCLC.

### Ethics statement

This is a retrospective real-world study approved by the Sir Run Run Shaw Hospital, Zhejiang University School of Medicine Ethics Committee (20211215-31) and conducted according to the principles of the Declaration of Helsinki. The requirement for informed consent was waived by the Sir Run Run Shaw Hospital, Zhejiang University School of Medicine Ethics Committee in view of the retrospective nature of the study.

### Study design

Clinical information was collected, including but not limited to demographic information, Smoking history, ECOG score, histological types, stages, metastases sites, current or subsequent mutations, anti-angiogenic treatments, 1st, 2nd, and 3rd line TKIs, chemotherapy information, radiotherapy information, immunotherapies, PFS, OS, and adverse events. The OS of patients receiving anti-angiogenic agents or not throughout the treatment course was compared, and stratified by patients with or without liver metastases or brain metastases.

### Statistical analysis

Data were analyzed using the IBM SPSS Statistics 25 software. The corresponding 95% confidence interval (95% CI) was calculated, and p values of < 5% (p < 0.05) were considered statistically significant.

Kaplan–Meier survival curves were constructed for overall survival and progression-free survival while compared using a stratified log-rank test. Hazard Ratio (HR) and associated 95% CIs were calculated by Cox proportional hazard analysis.

## Supplementary Information

Supplementary Tables.

Supplementary Information.

## Data Availability

All data generated or analyzed during this study are included in this published article (and its Supplementary Information files).

## References

[1] Siegel RL, Miller KD, Fuchs HE, Jemal A (2021). Cancer statistics, 2021. CA Cancer J. Clin..

[2] Bray F (2018). Global cancer statistics 2018: GLOBOCAN estimates of incidence and mortality worldwide for 36 cancers in 185 countries. CA Cancer J. Clin..

[3] Li XY, Lin JZ, Yu SH (2020). Front-line therapy in advanced non-small cell lung cancer with sensitive epidermal growth factor receptor mutations: A network meta-analysis. Clin. Ther..

[4] Hsu WH, Yang JC, Mok TS, Loong HH (2018). Overview of current systemic management of EGFR-mutant NSCLC. Ann. Oncol..

[5] Mok TS (2009). Gefitinib or carboplatin-paclitaxel in pulmonary adenocarcinoma. N. Engl. J. Med..

[6] Zhou C (2011). Erlotinib versus chemotherapy as first-line treatment for patients with advanced EGFR mutation-positive non-small-cell lung cancer (OPTIMAL, CTONG-0802): A multicentre, open-label, randomised, phase 3 study. Lancet Oncol..

[7] Park K (2016). Afatinib versus gefitinib as first-line treatment of patients with EGFR mutation-positive non-small-cell lung cancer (LUX-Lung 7): A phase 2B, open-label, randomised controlled trial. Lancet Oncol..

[8] Cheng Y (2021). Safety and efficacy of first-line dacomitinib in Asian patients with EGFR mutation-positive non-small cell lung cancer: Results from a randomized, open-label, phase 3 trial (ARCHER 1050). Lung Cancer.

[9] Soria JC (2018). Osimertinib in untreated EGFR-mutated advanced non-small-cell lung cancer. N. Engl. J. Med..

[10] Ramalingam SS (2020). Overall survival with osimertinib in untreated, EGFR-mutated advanced NSCLC. N. Engl. J. Med..

[11] Sheng M (2016). Comparison of clinical outcomes of patients with non-small-cell lung cancer harbouring epidermal growth factor receptor exon 19 or exon 21 mutations after tyrosine kinase inhibitors treatment: A meta-analysis. Eur. J. Clin. Pharmacol..

[12] Yang JC (2015). Afatinib versus cisplatin-based chemotherapy for EGFR mutation-positive lung adenocarcinoma (LUX-Lung 3 and LUX-Lung 6): Analysis of overall survival data from two randomised, phase 3 trials. Lancet Oncol..

[13] Herbst RS (2011). Efficacy of bevacizumab plus erlotinib versus erlotinib alone in advanced non-small-cell lung cancer after failure of standard first-line chemotherapy (BeTa): A double-blind, placebo-controlled, phase 3 trial. Lancet.

[14] Seto T (2014). Erlotinib alone or with bevacizumab as first-line therapy in patients with advanced non-squamous non-small-cell lung cancer harbouring EGFR mutations (JO25567): An open-label, randomised, multicentre, phase 2 study. Lancet Oncol..

[15] Saito H (2019). Erlotinib plus bevacizumab versus erlotinib alone in patients with EGFR-positive advanced non-squamous non-small-cell lung cancer (NEJ026): Interim analysis of an open-label, randomised, multicentre, phase 3 trial. Lancet Oncol..

[16] Yamamoto N (2021). Erlotinib plus bevacizumab vs erlotinib monotherapy as first-line treatment for advanced EGFR mutation-positive non-squamous non-small-cell lung cancer: Survival follow-up results of the randomized JO25567 study. Lung Cancer.

[17] Nakagawa K (2019). Ramucirumab plus erlotinib in patients with untreated, EGFR-mutated, advanced non-small-cell lung cancer (RELAY): A randomised, double-blind, placebo-controlled, phase 3 trial. Lancet Oncol..

[18] Zhou Q (2021). Bevacizumab plus erlotinib in Chinese patients with untreated, EGFR-mutated, advanced NSCLC (ARTEMIS-CTONG1509): A multicenter phase 3 study. Cancer Cell.

[19] Gridelli C (2018). Safety and efficacy of bevacizumab plus standard-of-care treatment beyond disease progression in patients with advanced non-small cell lung cancer: The AvaALL randomized clinical trial. JAMA Oncol..

[20] Garon EB (2014). Ramucirumab plus docetaxel versus placebo plus docetaxel for second-line treatment of stage IV non-small-cell lung cancer after disease progression on platinum-based therapy (REVEL): A multicentre, double-blind, randomised phase 3 trial. Lancet.

[21] Lee CK (2015). Impact of specific epidermal growth factor receptor (EGFR) mutations and clinical characteristics on outcomes after treatment with EGFR tyrosine kinase inhibitors versus chemotherapy in EGFR-mutant lung cancer: A meta-analysis. J. Clin. Oncol..

[22] Landre T (2020). First-line angiogenesis inhibitor plus erlotinib versus erlotinib alone for advanced non-small-cell lung cancer harboring an EGFR mutation. J. Cancer Res. Clin. Oncol..

[23] Sun L, Ma JT, Zhang SL, Zou HW, Han CB (2015). Efficacy and safety of chemotherapy or tyrosine kinase inhibitors combined with bevacizumab versus chemotherapy or tyrosine kinase inhibitors alone in the treatment of non-small cell lung cancer: A systematic review and meta-analysis. Med. Oncol..

[24] Deng Z, Qin Y, Liu Y, Zhang Y, Lu Y (2021). Role of antiangiogenic agents combined with EGFR tyrosine kinase inhibitors in treatment-naive lung cancer: A meta-analysis. Clin. Lung Cancer.

[25] Yu X, Sheng J, Pan G, Fan Y (2021). Real-world utilization of EGFR TKIs and prognostic factors for survival in EGFR-mutated non-small cell lung cancer patients with brain metastases. Int. J. Cancer.

[26] Castañón E (2015). Impact of epidermal growth factor receptor (EGFR) activating mutations and their targeted treatment in the prognosis of stage IV non-small cell lung cancer (NSCLC) patients harboring liver metastasis. J. Transl. Med..

[27] Cantelmo AR (2020). Angiogenesis inhibition in non-small cell lung cancer: A critical appraisal, basic concepts and updates from American Society for Clinical Oncology 2019. Curr. Opin. Oncol..

[28] Reungwetwattana T (2018). CNS response to osimertinib versus standard epidermal growth factor receptor tyrosine kinase inhibitors in patients with untreated EGFR-mutated advanced non-small-cell lung cancer. J. Clin. Oncol..

[29] Kumar A, Petri ET, Halmos B, Boggon TJ (2008). Structure and clinical relevance of the epidermal growth factor receptor in human cancer. J. Clin. Oncol..

[30] Li WQ, Cui JW (2020). Non-small cell lung cancer patients with ex19del or exon 21 L858R mutation: Distinct mechanisms, different efficacies to treatments. J. Cancer Res. Clin. Oncol..

[31] Reguart N, Remon J (2015). Common EGFR-mutated subgroups (Del19/L858R) in advanced non-small-cell lung cancer: Chasing better outcomes with tyrosine kinase inhibitors. Future Oncol..

[32] Offin M (2019). Tumor mutation burden and efficacy of EGFR-tyrosine kinase inhibitors in patients with EGFR-mutant lung cancers. Clin. Cancer Res..

[33] Tsai MF (2015). EGFR-L858R mutant enhances lung adenocarcinoma cell invasive ability and promotes malignant pleural effusion formation through activation of the CXCL12-CXCR4 pathway. Sci. Rep..

