# Supplementary material for: The benefit of anti-angiogenic therapy in EGFR exon 21 L858R mutant non-small cell lung cancer patients: a retrospective study
Source: Sci Rep. 2022 Aug 26;12:14624. doi: 10.1038/s41598-022-18889-z (PMC9418331; doi:10.1038/s41598-022-18889-z)
Supplement: Supplementary file 1 — Supplementary Tables. [file 41598_2022_18889_MOESM1_ESM.docx]

**Supplemental Table 1** Clinical characteristics for liver metastatic patients

| Patients, No. (%) | | | | |
| --- | --- | --- | --- | --- |
|  | Liver metastasis | | Liver metastasis free | |
| Characteristic | Cohort A | Cohort B | Cohort A | Cohort B |
| No. of patients | 9 | 16 | 49 | 85 |
| Median age, years(range) | 69(48-75) | 62(50-82) | 66(47-84) | 64(43-94) |
| Gender |  |  |  |  |
| Male | 5(56%) | 5(31%) | 28(57%) | 36(42%) |
| Female | 4(44%) | 11(69%) | 21(42%) | 49(58%) |
| Smoking history |  |  |  |  |
| Never | 6(67%) | 14(88%) | 38(78%) | 63(74%) |
| Former | 3(33%) | 2(13%) | 11(22%) | 22(26%) |
| ECOG |  |  |  |  |
| 0 | 2(22%) | 11(69%) | 30(61%) | 57(67%) |
| 1 | 7(78%) | 5(31%) | 19(39%) | 28(33%) |
| Stage |  |  |  |  |
| Ⅲ | 0 | 0 | 4(8%) | 6(7%) |
| IV | 9(100%) | 16(100%) | 45(92%) | 79(93%) |
| Concurrent mutations |  |  |  |  |
| Yes | 5(56%) | 3(19%) | 18(37%) | 20(24%) |
| No | 4(44%) | 13(81%) | 31(63%) | 65(76%) |

**Supplemental Table 2** Clinical characteristics for brain metastatic patients

| Patients, No. (%) | | | | | | |
| --- | --- | --- | --- | --- | --- | --- |
|  | Brain metastasis with radiotherapy | | Brain metastasis without radiotherapy | | Brain metastasis free | |
| Characteristic | Cohort A | Cohort B | Cohort A | Cohort B | Cohort A | Cohort B |
| No. of patients | 14 | 10 | 4 | 21 | 40 | 70 |
| Median age, years(range) | 62(51-72) | 66(57-82) | 57(53-68) | 63(43-84) | 66(47-84) | 64(44-94) |
| Gender |  |  |  |  |  |  |
| Male | 6(43%) | 3(30%) | 4(100%) | 8(38%) | 23(57%) | 30(43%) |
| Female | 8(57%) | 7(70%) | 0 | 13(62%) | 17(43%) | 40(57%) |
| Smoking history |  |  |  |  |  |  |
| Never | 10(71%) | 8(80%) | 3(75%) | 17(81%) | 31(77%) | 52(74%) |
| Former | 4(29%) | 2(20%) | 1(25%) | 4(19%) | 9(23%) | 18(26%) |
| ECOG |  |  |  |  |  |  |
| 0 | 8(57%) | 6(60%) | 2(50%) | 11(52%) | 22(55%) | 51(73%) |
| 1 | 6(43%) | 4(40%) | 2(50%) | 10(48%) | 18(45%) | 19(27%) |
| Stage |  |  |  |  |  |  |
| Ⅲ | 0 | 0 | 0 | 0 | 4(10%) | 6(9%) |
| IV | 14(100%) | 10(100%) | 4(100%) | 21(100%) | 36(90%) | 64(91%) |
| Concurrent mutations |  |  |  |  |  |  |
| Yes | 4(29%) | 2(20%) | 2(50%) | 6(29%) | 17(42%) | 15(21%) |
| No | 10(71%) | 8(80%) | 2(50%) | 15(71%) | 23(58%) | 55(77%) |

**Supplemental Table 3** Clinical characteristics for patients with early-line anti-angiogenesis

| Clinical Characteristics | | |
| --- | --- | --- |
|  | Patients, No. (%) |  |
| Characteristic | Cohort C | Cohort B |
| No. of patients | 26 | 101 |
| Median age, years(range) | 66(47-84) | 64(43-94) |
| Gender |  |  |
| Male | 16(61%) | 41(41%) |
| Female | 10(39%) | 60(59%) |
| Smoking history |  |  |
| Never | 19(73%) | 77(76%) |
| Former | 7(27%) | 24(24%) |
| ECOG |  |  |
| 0 | 15(58%) | 68(67%) |
| 1 | 11(42%) | 33(33%) |
| Stage |  |  |
| Ⅲ | 1(4%) | 6(7%) |
| IV | 25(96%) | 95(94%) |
| Concurrent mutations |  |  |
| Yes | 10(39%) | 23(23%) |
| No | 16(61%) | 78(77%) |
| Brain metastases |  |  |
| Yes | 4(15%) | 31(31%) |
| No | 22(85%) | 70(69%) |
| Liver metastases |  |  |
| Yes | 3(12%) | 16(16%) |
| No | 23(88%) | 85(84%) |
